# The adverse impact of excessive smartphone screen-time on sleep quality among young adults: A prospective cohort

**DOI:** 10.5935/1984-0063.20200114

**Published:** 2021

**Authors:** Daneyal Arshad, Usaid Munir Joyia, Sadaf Fatima, Noor Khalid, Anser Ikram Rishi, Naimat Ullah Abdul Rahim, Syed Faheem Bukhari, Gulfam Khan Shairwani, Ahmed Salmaan

**Affiliations:** Rawalpindi Medical University, Rawalpindi, Pakistan.

**Keywords:** Sleep Hygiene, Sleep Initiation and Maintenance Disorders, Smartphone, Screen Time

## Abstract

**Introduction:**

Problematic over usage of smartphones has led to various deteriorating effects including poor sleep quality. Screen exposure, especially near bedtime, directly leads to poor sleep quality. We aimed to measure smartphone screen-time (ST) statistics of the participants directly using a smartphone application. Furthermore, we aimed to assess sleep quality using the Pittsburgh sleep quality index (PSQI), and to investigate the association between ST & PSQI.

**Material and Methods:**

This descriptive cohort study was conducted among 280 students of MBBS at Rawalpindi Medical University for a period of 1 month (30 days). Physically healthy students who owned Android smartphones were included in the study. Students with diagnosed sleep disorders and students taking sleep medication were excluded from the study. ST was recorded using a smartphone application. Sleep quality was assessed at the end of 30 days using the PSQI questionnaire. Data entry and analysis was done using SPSS v23.0.

**Results:**

Total and mean ST were calculated for every participant. The mean screen time of 242 individuals was 147.50±51.09 hours. The mean PSQI score was 6.68±2.3. 65.70% of the participants had a poor sleep quality (PSQI>5). Pearson’s correlation revealed that long total ST was associated with decreased sleep quality (R=0.356, p<0.001).

**Conclusion:**

Our findings are in accordance with previous scientific literature largely based on self-reported ST measurements and affirm that excessive ST deteriorates sleep quality and hence has numerous adverse physical and psychological manifestations.

## INTRODUCTION

The advent of smartphones took the world by storm, and smartphones have nearly become ubiquitous, especially in the developed and developing countries. This has led, inevitably, to smartphone dependency and excessive usage. Problematic over usage of smartphones has manifested as an array of deteriorating effects including poor sleep quality. Smartphone usage, especially near bedtime, worsens one’s experience of sleep and directly leads to poor sleep quality. The encyclopedia of behavioral medicine defines sleep quality as one’s satisfaction of the sleep experience, integrating aspects of sleep initiation, sleep maintenance, quantity, and refreshment upon awakening^1^.

Smartphone screens emit blue light in the short wavelength region (380nm to 495nm). This blue light is considered to be responsible for suppressing the production of melatonin hormone, which is one of the key components in the regulation of the sleep-wake cycle. This leads to sleep disturbances and contributes to poor sleep quality^2^.

Sleep disturbances have drastic adverse effects on physical and mental health. Poor sleep quality has been reported to increase the risk of weight gain, obesity, metabolic syndrome, hypertension, glucose intolerance, and diabetes^3, 4, 5, 6^. Sleep deprivation negatively affects cognitive functions^7^ and can also lead to depression, stress, and anxiety^8, 9^.

This study aimed to directly measure the duration for which a person uses the smartphone, i.e., screen time (ST) and to find an association between ST and sleep quality, quantitatively measured by Pittsburgh sleep quality index (PSQI). There is a multitude of studies that have been conducted among students to explore the prevalence of decreased sleep quality^10, 11, 12^, but there is a scarcity of studies investigating the direct associations between sleep quality and smartphone usage.

Thus, we aim to contribute to this knowledge gap. Recognizing the adverse effects of excessive phone usage on sleep quality, and subsequently on the mental and physical health of the subjects would necessitate the importance of timely interventions and remedies.

## MATERIAL AND METHODS

### Participants’ recruitment and characteristics

This descriptive cohort study was conducted among 280 students of MBBS at Rawalpindi Medical University, Rawalpindi, Pakistan, for a period of one month from 8^th^ July 2019 to 7^th^ August 2019 (30 days). Ethical approval was taken from the ethical review board prior to participant recruitment and data collection. Participants were sampled using non-randomized consecutive sampling. Physically healthy students who owned an android smartphone were included in the study. Students with diagnosed insomnia or other sleep disorders and students taking sleep medication were excluded from the study. Consent was obtained from all participants and the aims and procedures of the study were explained to them.

### Study procedures and data collection: screen time

Participants were instructed to download and install an Android smartphone application. This application tracked ST duration and reported it in the form of a bar chart. The bar chart displayed daily usage statistics of seven days separately at a given time. The usage duration data could also be exported as an *Excel Spreadsheet* (.xlsx).

The participants were approached once every week (for a total of 4 weeks) and were instructed to send a screenshot of the bar chart along with the *Excel Spreadsheet*. Data of two additional days were also collected to make a total of 30 days. Participants for whom the complete data of 30 days was available were selected for entry and analysis. Participants with missing or incomplete data were excluded.

### Study procedures and data collection: sleep quality

Sleep quality was assessed at the end of 30 days using the Pittsburgh sleep quality index (PSQI) questionnaire. Poor sleep quality was defined as a global PSQI score>5. PSQI scores were entered as a scale variable while measuring the association with ST. An online form version of the original questionnaire was sent to the participants. Global PSQI score and total ST were assessed for possible associations.

### Analytic approach

Data entry and analysis was done using IBM SPSS Statistics for Windows, version 23.0 (IBM Corp., Armonk, N.Y., USA). Means and standard deviations were used to report normally distributed variables. Pearson’s chi-square test, independent samples T-test, Pearson’s correlation test, and linear regression were performed.

## RESULTS

The study included 280 participants, amongst which 48.93% (n=137) were males and 51.07% (n=143) were females. The mean age was 21.89±1.73 years. Complete data was available for 242 participants (males: 47.93% (n=116), females: 52.06% (n=126)). 27 had missing data, 11 dropped out of the study for various reasons (change of consent: 8, uninstalled the application: 3). For each of these 242 participants, total and average ST were calculated. Global PSQI scores were also entered for each participant.

### Prevalence of poor sleep quality:

65.70% (n=159) of participants had poor sleep quality (PSQI>5) and 34.29% (n=83) had good sleep quality. The mean PSQI score was 6.68±2.3. Mean PSQI scores in participants with poor and good sleep qualities were 7.98±1.66 and 4.18±0.76, respectively (*p*≤0.001).

Males were prone to have poorer sleep quality than females. This difference was elicited by chi-square test. 72.41% (n=84) of the males had poor sleep quality. In contrast, 59.52% (n=75) females had poor sleep quality (*p*= 0.042).

### Screen-time statistics

The mean ST of 30 days was 147.50±51.09 hours. The maximum ST was 247.03 hours and the minimum ST was 22.50 hours.. There was a significant difference in the mean ST between the genders, as shown in Table 1. Mean ST for females (140.38±49.38) was lesser than for males (155.23±51.99) (*p*=0.024).

**Table 1:** Demographics, screen-time and PSQI statistics.

		Males		Females	*p*-value
**No. of participants^a^ (%)**		116 (47.9%)		126 (52.1%)	
**Mean age (years)**			21.79±1.76		
		21.85±1.67		21.73±1.83	0.611
**Mean screen-time (hours)**			147.50±51.09		
		155.23±51.99		140.38±49.38	0.024*
**Mean PSQI score**			6.68±2.3		
		6.80±2.16		6.57±2.42	0.438
**Mean PSQI score in**	Poor sleep quality PSQI>5)		7.98±1.66		<0.001*
	Good sleep quality (PSQI≤5)		4.18±0.76		
**Sleep quality**	Poor sleep quality PSQI>5)	84 (72.41%^b^)		75 (59.52%^c^)	0.042*
	Good sleep quality (PSQI≤5)	32 (27.59%^b^)		51 (40.48%^c^)	
**Mean screen-time in**	Poor sleep quality PSQI>5)		152.63±56.74		0.030*
	Good sleep quality (PSQI≤5)		137.67±36.34		

^a^ After excluding those with missing data;

^b^ % of males;

^c^ % of females;

*Significant (p<0.05).

Mean ST for participants with poor sleep quality was 152.63±56.74, while for those with good sleep quality was 137.67±36.34 (*p*=0.030). These results described above have been summarized in Table 1.

### Association of screen time and sleep quality

Pearson’s correlation and simple linear regression revealed a strong positive association of ST and PSQI score (R=0.356, *p*≤0.001). R-squared change of 0.127 showed that 12.7% of the variance in sleep quality can be attributed to ST. These results are represented in Table 2.

**Table 2:** Correlation and linear regression results.

	R	R Squared Change	PSQI Score Standardized Beta Coefficient	F Change	P Value
Total Screen-time	0.356*	0.127	0.356*	34.86	<0.0010*

* Significant (p<0.001)

## DISCUSSION

Sleep is crucial for maintaining an adequate and healthy lifestyle. It can be considered as a maintenance and repair period of the body during which the metabolites that have accumulated throughout the day are cleared, and mental stress and anxiety are relieved. Hence it goes without saying that anything that disturbs the sleep pattern impairs these protective and repair functions, and leads to physical as well as mental manifestations^13, 14^.

The regulation of sleep is a multi-factorial process. The pineal gland, and its hormone melatonin, play a major role in the regulation of the circadian rhythm and sleep initiation. Melatonin is produced at night in a dark environment. Short-wavelength blue light (380-495nm) is known to adversely affect melatonin production^2, 15^. This dose-dependent suppression of melatonin production in turn leads to sleep disruption^2^. Poor sleep quality has been associated with excessive weight gain and obesity^3, 4, 16^. Poor sleep quality may also be associated with negative academic performance^17^. Insufficient sleep has also been linked with an increased risk of developing metabolic syndrome^5^ and diabetes mellitus^18^. Shorter sleep duration is associated with an increased risk for hypertension, especially in individuals under the age of 65^6^. It also negatively affects attention, working memory, and other cognitive functions^7^. Various studies have also reported an association of poor sleep quality with depression and anxiety^8, 9^.

Pittsburgh sleep quality index (PSQI) is a reliable and validated self-administered questionnaire, which assesses sleep quality over a period of thirty (30) days. It defines poor sleep quality as a global PSQI score>5 (89.6% sensitivity and 86.5% specificity). In accordance with this, the major proportion of our participants had poor sleep quality (65.70%, n=159). This indicates a high prevalence of poor sleep quality. Many similar studies conducted among students have shown this trend. A 2015 study conducted among medical students of Karachi, Pakistan, reported that 39.5% of its subjects had poor sleep quality^10^. In another study conducted in 2018 across 11 educational institutions of Pakistan, 59.03% of the subjects were found out to have poor sleep quality^11^.

The mean ST of the participants was 147.50±51.90 hours and the maximum ST recorded was 247.03 hours. To put these two numbers into perspective, these equate to 6.15 and 10.29 days per month of phone usage, respectively. Indeed, these numbers are alarmingly high. Furthermore, ST came out to be strongly positively correlated with the PSQI score. PSQI score for the maximum-recorded ST was 11, indicating a poor sleep quality. Results reveal that at least a 12.7% variance in sleep quality is explained by ST.

A self-reported questionnaire-based study conducted among Chinese medical students reported that excessive ST was significantly associated with poor sleep quality. The participants, which included 4,915 college students, reported time spent on computers and watching TV/video games. This ST was categorized as ≤2 hours/day and >2 hours/day. Data analysis revealed that participants in the latter category had poorer sleep quality (OR=1.32, 95% CI: 1.06-1.65)^19^.

Another study conducted at Wuhan University also used similar methods to find out the association between ST with sleep quality, while investigating other factors too. This study also used self-reported ST categorized as ≤2 hours/day and >2 hours/day and found that participants in the former category were less likely to have poor sleep quality (OR=1.11, 95% CI: 0.77-1.58)^20^.

A cross-sectional study conducted on 1,674 US adults investigated the association of sedentary time and ST with sleep outcomes. It categorized participants’ self-reported ST into 4 categories: ≤2.0h/day; 2.1-4.0h/day; 4.1-6.0h/day; >6h/day. Statistical analysis revealed that participants in category 4 (>6h/ day) had 3.1-fold higher odds of reporting sleep difficulties compared to those in the first category^21^.

All three of these studies confirm the findings of our study. However, these studies utilize self-reported ST statistics, which can be different from actual usage duration. People tend to under-report their usage times, as observed by a study conducted among university students in Taiwan, in 2015, which found out that their participants grossly underestimated their smartphone usage duration^22^. Our study, therefore, removes this participant bias of under or overreporting their ST by directly measuring it as a continuous variable.

Another study conducted among US adults also measured ST directly using an android smartphone application. This study also found out that poor sleep quality (PSQI>5) and longer ST were significantly associated with each other. However, this particular observation was based on the data of 56 participants (out of 653 participants that had completed the core survey) that had complete ST and PSQI data^23^. Our study incorporates a relatively larger number of participants (n=242) to establish this association.

Our findings highlight the necessity of curbing excessive smartphone usage as well as exploring remedies to ameliorate poor sleep quality. Using blue light filters for screens can attenuate the harmful effects of blue light on sleep^24^. Bedtime phone usage restriction has also been found beneficial to increase sleep duration^25^. The use of melatonin supplements is proposed to increase sleep duration and decrease sleep latency^26^.

This study successfully concludes, in accordance with previous studies done on the subject, that excessive smartphone usage is one of the factors contributing to adverse sleep quality. To evaluate the remaining variance in decreased sleep quality was beyond the scope of this study. The remaining variance in sleep quality may be attributed to other factors such as anxiety and emotional stress, as reported by other studies^27, 28^.

It is also important to state that these results alone are not enough to establish causation. Further researches and experimental study designs are needed to establish temporality and cause-effect relationships. The Bradford Hill criteria can be used as a guideline in this regard^29^. Furthermore, we could not account for ST other than smartphones, which includes laptops and television screens. A multivariate analysis accounting for ST across all devices as well as taking into account other factors that infuence sleep quality would provide a more inclusive and comprehensive overview.

## CONCLUSIONS

The findings of our study are in accordance with past researches done on the subject and reaffirm that young adults spend a significant amount of time using smartphones, which directly leads to decreased sleep quality. This subsequently predisposes them to multiple adverse physical as well as psychological health outcomes. It is therefore recommended to take measures to decrease ST, particularly in the hours prior to sleeping. The use of blue light filters for screens should be advised, since blue light decreases sleep quality.
